# Endovascular treatment of lower limb acute DVT: current trends and future directions

**DOI:** 10.1186/s42155-024-00495-x

**Published:** 2024-11-26

**Authors:** Francesco Siciliano, Edoardo Ronconi, Tommaso Rossi, Federica Fanelli, Miltiadis Krokidis, Pasqualino Sirignano, Michele Rossi, Marcello Andrea Tipaldi

**Affiliations:** 1 Department of Surgical and Medical Sciences and Translational Medicine School of Medicine and Psychology, “Sapienza” - University of Rome, Rome, Italy; 2 Department of Radiology, Sant’Andrea University Hospital La Sapienza, Rome, Italy; 3 Department of Radiological Sciences, Oncology and Pathology, Policlinico Umberto I Hospital, Sapienza University of Rome, Viale del Policlinico 155 Sapienza, Rome, 00161 Italy; 4 School of Medicine, National and Kapodistrian University of Athens Areteion Hospital , 76 Vas. Sophias Ave, 11528 Athens, Greece; 5 Vascular and Endovascular Surgery Unit, Sant’Andrea Hospital of Rome, Department of General and Specialistic Surgery, “Sapienza” University of Rome, 00189 Rome, Italy

**Keywords:** Acute vein thrombosis, Deep vein thrombosis, Endovascular thrombus removal, Catheter-directed thrombolysis

## Abstract

**Aim of the study:**

This systematic review aims to evaluate the efficacy, safety, and comparative outcomes of endovascular treatments for acute lower limb deep vein thrombosis (DVT), including catheter-directed thrombolysis (CDT), pharmacomechanical thrombectomy (PMT), mechanical thrombectomy, and venous stenting, drawing insights from a diverse range of studies.

**Materials and methods:**

A comprehensive literature search identified 33 relevant studies, including randomized controlled trials, cohort studies, systematic reviews, and case reports. Data extraction focused on study design, intervention type, outcome measures, and follow-up duration.

**Results:**

Catheter-directed thrombolysis demonstrates promising results in enhancing venous patency and reducing post-thrombotic syndrome, with careful patient selection being crucial. Pharmacomechanical and mechanical thrombectomy devices offer immediate and long-term benefits, emphasizing individualized patient care. Venous stenting serves as a crucial adjunctive therapy, particularly in cases of residual venous obstruction, though further research is needed for optimal patient selection and long-term outcomes. Timing and selection of endovascular interventions remain critical considerations, necessitating multidisciplinary approaches and ongoing research.

**Conclusion:**

This review provides valuable insights for clinicians and researchers, guiding evidence-based decision-making and shaping future research directions in the dynamic field of endovascular interventions for acute lower limb DVT.

## Introduction

Acute deep vein thrombosis (DVT) of the lower extremities is a prevalent vascular disorder characterized by the sudden formation of blood clots within the deep veins, often associated with significant morbidity and mortality if left untreated [1]. Acute DVT refers to the presence of symptoms for less than 14 days or for which imaging studies indicate that thrombosis occurred within the previous 14 days [2]. Prompt and effective management of acute DVT is imperative to prevent potential life-threatening complications, such as pulmonary embolism and the development of post-thrombotic syndrome [3]. Historically, anticoagulation therapy has been the primary approach for the treatment of acute DVT, aiming to halt clot propagation and reduce the risk of embolization [4]. However, advancements in endovascular techniques have introduced new possibilities for the rapid and targeted treatment of acute DVT.

European Society for Vascular Surgery (ESVS) and the American Society of Interventional Radiology (SIR), emphasizing a comprehensive approach to management. Current recommendations advocate for a combination of anticoagulation therapy, thrombolysis, and mechanical thrombectomy depending on the severity and location of the thrombus. Anticoagulation with low molecular weight heparin (LMWH) or direct oral anticoagulants (DOACs) remains the cornerstone of initial therapy to prevent clot extension and embolization. In cases of extensive or severe DVT, especially involving the iliofemoral veins, endovascular techniques such as catheter-directed thrombolysis (CDT) or percutaneous mechanical thrombectomy (PMT) may be considered to expedite thrombus resolution and reduce long-term complications such as post-thrombotic syndrome (PTS) and recurrent thrombosis (ESVS, SIR) [5, 6].

Endovascular interventions, including catheter-directed thrombolysis (CDT), pharmacomechanical thrombolysis (PMT), and percutaneous mechanical thrombectomy (PMTB), have emerged as promising strategies to achieve prompt clot dissolution and venous patency restoration in patients with acute DVT [7, 8]. These techniques provide the potential for more efficient clot removal and faster symptom resolution, potentially reducing the incidence of post-thrombotic complications and improving overall patient outcomes [9]. Nonetheless, a comprehensive assessment of the available evidence regarding the efficacy, safety, and comparative effectiveness of these endovascular approaches for acute DVT is essential to guide clinical decision-making.

This systematic review aims to comprehensively evaluate and synthesize the existing literature on the endovascular treatment of acute DVT of the lower limb. By rigorously analyzing published studies and clinical trials, we seek to delineate the current evidence surrounding endovascular interventions, providing insights into their efficacy, safety, and comparative outcomes in the management of acute lower limb DVT. Ultimately, this review strives to inform clinicians, researchers, and healthcare practitioners regarding the optimal utilization of endovascular treatments in the acute phase of DVT, promoting evidence-based care and influencing future research directions.

## Materials and methods

### Literature search strategy

A systematic and comprehensive literature search was conducted to identify relevant studies pertaining to the endovascular treatment of deep vein thrombosis (DVT) in the lower limb. Databases including PubMed and Cochrane Library were searched up to [insert end date of the search] using a predefined search strategy. The search strategy employed a combination of keywords and MeSH terms, including “deep vein thrombosis,” “lower limb,” “endovascular treatment,” “catheter-directed thrombolysis,” “pharmacomechanical thrombolysis,” and “percutaneous mechanical thrombectomy.”

### Study selection and eligibility criteria

Inclusion and exclusion criteria were defined to select relevant studies for this review. Studies included were required to focus on the endovascular treatment of DVT in the lower limb. Randomized controlled trials (RCTs), prospective and retrospective cohort studies, case–control studies, case reports and systematic reviews/meta-analyses were considered for inclusion. Studies involving adult human subjects and published in English were included. Animal studies and conference abstracts were excluded.

### Data extraction

Two reviewers conducted the initial screening of titles and abstracts based on the eligibility criteria. Full-text articles of potentially relevant studies were assessed for further eligibility. Disagreements were resolved through discussion and consensus or by consulting a third reviewer when necessary. Data extraction was performed using a standardized data extraction form, including details on study design, sample size, intervention type, outcome measures, and follow-up duration.

### Data synthesis

A narrative synthesis of the included studies was performed to summarize the findings related to the endovascular treatment of DVT in the lower limb. The synthesis focused on intervention types, efficacy outcomes (e.g., clot reduction, venous patency), safety outcomes (e.g., bleeding events), and follow-up durations.

## Results

### Inclusion and quality assessment

**Fig. 1:**
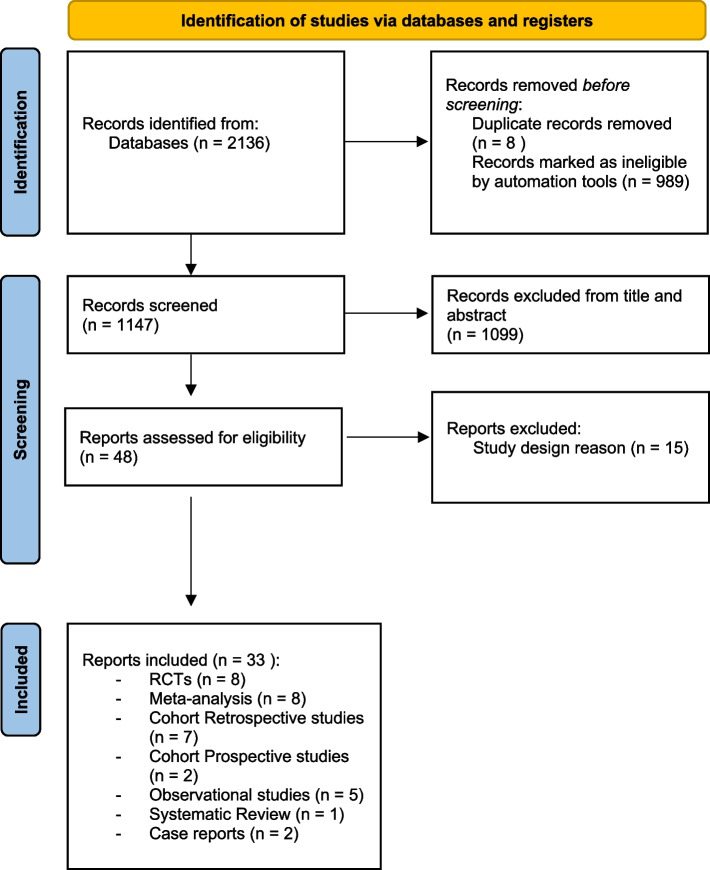
Prisma flow diagram

The initial search strategy resulted in a total of 2136 studies and, after applying exclusion criteria and screening title, abstract and full text, as for PRISMA guidelines [10], 33 studies were included, (Fig. 1).

**Fig. 2:**
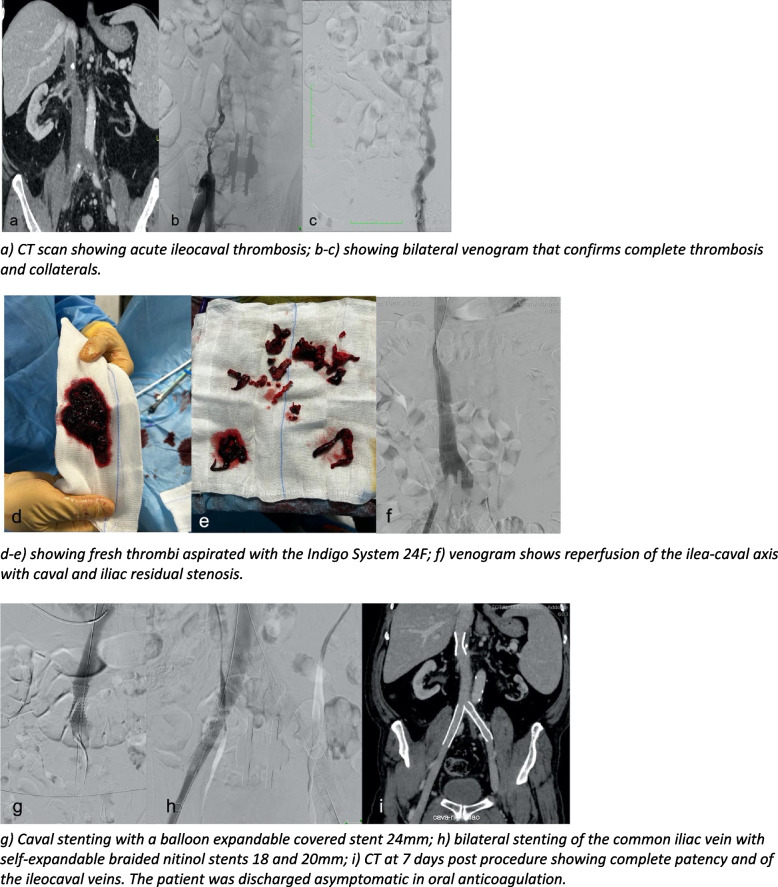
Showing a case of complex acute ileo-caval thrombosis treatment in a symptomatic patient with bilateral pain and legs swelling

The studies consisted of 8 meta-analysis, 8 randomised controlled trials (RCTs), 7 cohort retrospective studies, 2 cohort prospective studies, 5 observational studies, 1 systematic review and 2 case reports (Fig. 2).

1. Catheter-directed thrombolysis (Table 1).

**Table 1:** Summary of catheter-directed thrombolysis studies

Study	Population	Intervention	Main Outcomes	Key Findings
Enden et al. [6]	189 patients with iliofemoral DVT	CDT vs. control	- Oral anticoagulation within therapeutic range at 6 months: CDT 61.1%, Control 52.6%—Oral anticoagulation within therapeutic range at 24 months: CDT 65.4%, Control 50.0%—Reduction in PTS at 24 months: Absolute risk reduction of 14.4%—Iliofemoral patency at 6 months: Significantly improved in CDT group	- Higher oral anticoagulation rates in CDT group—Significant reduction in PTS at 24 months in CDT group—Improved iliofemoral patency in CDT group—20 bleeding complications in CDT group (3 major, 5 clinically relevant)
Notten et al. [8]	Not specified	Different treatment approaches for PTS	- Development of PTS: Reduced with ultrasound-accelerated CDT—Quality of life measures: No clinically relevant improvement	- Additional ultrasound-accelerated CDT reduced PTS, especially mild PTS—No clinically relevant improvement in quality of life
Zhu et al. [9]	Patients with lower extremity DVT	Ultrasound-guided CDT (Group A) vs. Non-guided CDT (Group B)	- Success rate with a single intubation attempt: Higher in Group A—Operation time: Significantly shorter in Group A—Incidence of hematoma at the intubation site: Significantly lower in Group A—Circumferential diameter before and after thrombolysis: Significantly better results in Group A—Venous patency of the affected limb: Significantly higher in Group A—Incidence of long-term PTS: Significantly lower in Group A	- Group A had higher success rate, shorter operation time, and fewer complications—Improved outcomes with ultrasound-guided CDT
Engelberger et al. [10]	48 patients with acute iliofemoral DVT	CDT vs. USAT	- PTS: Low incidence in both groups at 12 months—Quality of life measures: No significant difference between CDT and USAT groups	- Low incidence of PTS in both groups—Good quality of life in both groups—No significant difference between CDT and USAT groups
Zhang et al. [11]	386 patients with iliofemoral DVT	CDT alone vs. CDT with balloon angioplasty	- PTS severity at 2 years: No significant difference—Quality of Life (QoL): No significant difference—Impact based on duration of symptoms: Potential benefit of balloon angioplasty in subacute DVT	- No significant difference in PTS severity and QoL between groups—Potential benefit of balloon angioplasty in subacute DVT
Wang et al. [12]	Not specified	Case series studies on CDT complications	- Risk of major complications: Pooled risk of 3%—Risk of PE: Pooled risk of 0%—Risk of mortality: Pooled risk of 7%	- Low risk of complications with CDT—Very low risk of PE and rare mortality risk
Javed et al. [13]	Patients with acute proximal DVT	LCBIs vs. control	- Rate of PTS: Reduced with LCBIs—Major bleeding events: Increased with LCBIs	- LCBIs reduce PTS and moderate to severe PTS—Increased risk of major bleeding events
Lu et al. [13]	Patients with acute lower extremity DVT	CDT plus anticoagulation vs. anticoagulation alone	- Percentage patency of iliofemoral vein: Increased with CDT—Risk of PTS: Reduced with CDT—Risk of bleeding and PE events: Increased with CDT	- CDT improves venous patency and reduces PTS—Higher risk of bleeding and PE events with CDT—Longer hospital stay and higher charges in CDT group

Catheter-directed thrombolysis (CDT) has emerged as a promising and minimally invasive therapeutic approach for managing DVT, offering targeted delivery of thrombolytic agents directly to the clot site.

CDT involves the insertion of a catheter into the affected vein under imaging guidance, allowing for the administration of thrombolytic drugs, typically a thrombolytic agent such as tissue plasminogen activator (tPA), directly into the clot. This localized treatment aims to dissolve the clot, restore venous patency, and alleviate symptoms associated with DVT. The procedure has gained traction as an alternative to traditional systemic thrombolysis, which may be associated with an increased risk of bleeding.

The amalgamation of evidence derived from a series of clinical trials and systematic reviews investigating the efficacy of catheter-directed thrombolysis (CDT) and ultrasound-accelerated CDT for the management of deep vein thrombosis (DVT) offers significant insights into their effectiveness. According to the 2020 NICE guidelines, catheter-directed thrombolysis (CDT) is recommended as a therapeutic intervention for individuals diagnosed with iliofemoral deep vein thrombosis (DVT) who present with symptoms persisting for less than 14 days, exhibit a low risk of bleeding, maintain good functional status, and possess a life expectancy of one year or more [11].

Noteworthy studies [9, 12–19] collectively shed light on the positive impact of CDT in ameliorating venous patency and diminishing the incidence of post-thrombotic syndrome (PTS) in patients grappling with acute or subacute DVT. For instance, Enden et al. [9] randomized controlled trial spanning from January 2006 to December 2009, involving 209 patients, illustrated a significant reduction in the risk of PTS in those with iliofemoral DVT who underwent CDT. The absolute risk reduction of 14.4% at 24 months and enhanced short-term iliofemoral patency after 6 months underscored the efficacy of CDT, albeit with acknowledgment of a small additional risk of bleeding. Notten et al. [12] clinical trial, focusing on long-term follow-up of PTS patients, unveiled that supplementary ultrasound-accelerated catheter-directed thrombolysis could mitigate the risk of PTS, especially when employing the International Society of Thrombosis and Haemostasis (ISTH) definition. Despite these benefits, the study highlighted limited improvement in patients’ quality of life.

Zhu et al. [13] clinical trial comparing ultrasound-guided CDT with ultrasound non-guided CDT showcased the advantages of ultrasound guidance, including higher success rates, shorter operation times, lower incidence of hematoma, improved venous patency, and significantly lower rates of long-term PTS in the ultrasound-guided group. Engelberger et al. [14] BERNUTIFUL study, centered on acute iliofemoral DVT, demonstrated that a standardized catheter thrombolysis regimen followed by routine stenting resulted in low PTS incidence, good quality of life, and excellent patency rates with minimal postthrombotic vein lesions. Interestingly, the addition of intravascular ultrasound did not significantly impact the outcomes.

Lu Y et al. [18] conducted a meta-analysis comparing catheter-directed thrombolysis (CDT) with anticoagulation therapy alone for acute lower extremity deep vein thrombosis (DVT). CDT improved venous patency and reduced postthrombotic syndrome (PTS) but increased bleeding and pulmonary embolism (PE) risks. No significant differences were found in death or recurrent VTE events. CDT patients had longer hospital stays and higher charges. The decision to use CDT should consider patient risks and treatment benefits.

These findings collectively emphasize the promising benefits of CDT in enhancing venous patency and reducing PTS. However, the decision to employ CDT warrants careful consideration of individual patient risk profiles, weighing potential benefits against the heightened risk of bleeding and other complications. This underscores the imperative for ongoing research to refine techniques, minimize complications, and optimize DVT treatment strategies in clinical practice.

2. Pharmacomechanical Thrombectomy and Mechanical Thrombectomy (Table 2).

**Table 2:** Summary of Pharmacomechanical Thrombectomy and Mechanical Thrombectomy

Study	Population/Patients	Intervention	Main Outcome	Key Findings
Comerota AJ et al. [14]	692 patients with iliofemoral DVT	Pharmacomechanical catheter-directed thrombolysis (PCDT) vs. anticoagulation alone	Post-thrombotic syndrome (PTS), severity of PTS, venous disease-specific quality of life (QOL)	- PCDT led to significant improvements in leg pain, calf circumference, venous disease-specific QOL, and symptoms-specific QOL compared to anticoagulation alone. < br >—PCDT was not associated with significantly higher rates of major bleeding, recurrent venous thromboembolism, or death compared to anticoagulation alone
Kearon C et al. [15]	300 patients with femoral-popliteal DVT	Pharmacomechanical catheter-directed thrombolysis (PCDT) vs. no PCDT	Post-thrombotic syndrome (PTS)	- PCDT did not provide benefits in terms of preventing PTS, reducing leg pain, or improving leg swelling in patients with femoral-popliteal DVT. < br >—PCDT was associated with an increased risk of bleeding
Liu G et al. [16]	Not specified	AngioJet Rheolytic Thrombectomy (ART) system and catheter-directed thrombolysis (CDT)	Post-thrombotic syndrome (PTS), thrombus clearance	- Adjunctive thrombolysis and/or thrombectomy may have benefits for patients with lower extremity DVT. < br >—Thrombus clearance in calf veins may reduce the risk of proximal thrombosis recurrence and PTS. < br >—Selection of patients treated shortly after symptom onset appeared to yield better outcomes
Weinberg I et al. [17]	Not specified	Pharmacomechanical catheter-directed thrombolysis (PCDT)	Thrombus clearance, valvular reflux	- PCDT reduced thrombus burden but did not reduce the occurrence of valvular reflux. < br >—PCDT was associated with a reduced residual thrombus in the femoral vein, but this did not lead to improved clinical outcomes such as reduced post-thrombotic syndrome (PTS) or improved quality of life (QOL)
Vedantham S et al. [18]	Not specified	AngioJet Percutaneous Catheter-Directed Thrombolysis (PCDT)	Post-thrombotic syndrome (PTS), symptom relief, quality of life (QOL)	- AngioJet-PCDT showed short-term benefits in symptom relief and reducing PTS in patients with acute proximal DVT. < br >—These benefits did not persist over the long term. < br >—AngioJet-PCDT was associated with an increased risk of bleeding. < br >—The study emphasized the complexity of managing acute DVT and the need for further research to improve treatment strategies
Thukral S et al. [19]	300 patients with femoral-popliteal DVT	Pharmacomechanical catheter-directed thrombolysis (PCDT) vs. anticoagulation alone	Post-thrombotic syndrome (PTS), recurrent venous thromboembolism	- PCDT did not provide substantial benefits in terms of efficacy outcomes, either in the short or long term, for patients with femoral-popliteal DVT. < br >—PCDT was associated with an increased risk of bleeding. < br >—The authors recommended against the routine use of PCDT as the initial treatment for femoral-popliteal DVT
Cakir et al [20]	42 patients with iliofemoral-popliteal (proximal) DVT	Percutaneous aspiration thrombectomy	Venous patency rates, in-stent patency rates, clinical symptom scores	- Significantly higher patency rates at 1, 3, and 12 months compared to medical treatment group. < br >—Better in-stent patency rates with minimal incidents of in-stent thrombosis during 12-month follow-up. < br >—Improved clinical symptom scores compared to standard anticoagulant therapy, especially at 1, 3, and 12 months of follow-up
Kasirajan et al [21]	17 patients with extensive DVT	AngioJet rheolytic thrombectomy	Thrombus extraction, recurrence-free survival, clinical improvement	- Different levels of thrombus removal observed, with varying success rates. < br >—Clinical symptoms significantly improved in most patients. < br >—No complications directly linked to the AngioJet device reported. < br >—Recurrence-free survival rates: 81.6% at 4 months and 51.8% at 11 months
Loffroy et al [22]	30 patients with acute symptomatic DVT	Aspirex®S device	Technical success, primary patency, secondary patency, post-thrombotic syndrome	- Technical success rate: 100%, primary patency rate: 90%. < br >—Immediate clinical success achieved in 90% of patients. < br >—Secondary patency rate at mean follow-up of 22.3 months: 86.7%. < br >—No severe post-thrombotic syndrome (PTS) cases observed
Benarroch-Gampel J et al [23]	12 patients with lower extremity DVT	ClotTriever System	Symptom resolution, complication rate	- Successful clot evacuation achieved in all patients in a single session. < br >—100% of patients experienced symptom resolution before discharge with no significant complications reported. < br >—Sustained significant symptom relief reported at early follow-up
Crowner JR et al [24]	1 patient	INARI ClotTriever System	Symptom resolution, complication rate	- Single-session treatment of acute to subacute iliofemoral and caval DVT achieved without the need for prolonged infusion times or intensive care. < br >—Favorable outcomes in terms of initial symptom resolution and safety profile reported
Lopez et al [25]	Ten patients treated for iliofemoral or central DVT	Indigo continuous aspiration mechanical thrombectomy 8 system	Thrombus resolution, complications	- 60% technical success rate with the aspiration thrombectomy system. < br >—Complications were limited, and manageable outcomes were observed
Robertson et al [26]	16 patients with acute iliofemoral DVT	CMAT system using the Lightning 12 intelligent aspiration	Thrombus removal, complications	- All patients achieved > 70% thrombus reduction with minimal blood loss observed. < br >—Symptom resolution achieved in all patients before discharge with no postoperative complications reported
Li W et al [27]	Not specified	Adjuvant PMT during CDT	Venous patency, thrombolytic therapy duration, bleeding risk	- Adjuvant PMT with CDT resulted in higher rates of venous patency and thigh detumescence compared to CDT alone. < br >—Associated with reduced duration of thrombolytic therapy and lower incidence of major bleeding complications. < br >—Lower PTS incidence when PMT was used within the 2-year period after thrombolytic therapy
Wang W. et al [28]	1,323 patients with DVT	PMT alone or with CDT	Thrombus removal, thrombosis recurrence, complications	- High lysis rates, low thrombosis recurrence rates, and rare severe perioperative complications observed with PMT, either alone or in combination with CDT. < br >—Lower dose of thrombolytic drugs and shorter procedural times required compared to CDT alone. < br >—PMT, with or without CDT, considered an effective and safe alternative for treating DVT
Hu G et al [29]	Not specified	Percutaneous endovenous intervention (PEVI)	Risk of PTS and PE, venous patency, bleeding risk	- PEVI reduced the risk of PTS and PE, increased venous patency, and did not significantly increase bleeding risk. < br >—PEVI considered an effective and feasible approach for patients with acute LE-DVT

In the dynamic field of deep vein thrombosis (DVT) management, studies assess the efficacy and safety of diverse treatment approaches. The exploration of mechanical thrombectomy (MT) devices in the management of acute iliofemoral deep vein thrombosis (DVT) presents a multifaceted landscape, characterized by diverse outcomes from studies utilizing various devices such as AngioJet™, Aspirex™ S, Clottriever ® (Inari Medical Inc., CA, USA), Indigo® System CAT-8(Penumbra Inc., CA, USA), and Lightning® 12 (Penumbra Inc., CA, USA) that can be used alone or combining with clot-dissolving drugs in the Pharmacomechanical thrombectomy (PMT). Studies, from pharmacomechanical catheter-directed thrombolysis to AngioJet Rheolytic Thrombectomy (ART), explore treatment intricacies and their impact on post-thrombotic syndrome, quality of life, bleeding risks, and overall efficacy.

Comerota AJ et al. [30] ambitious study navigates the complex terrain of iliofemoral deep vein thrombosis (DVT) treatment, comparing pharmacomechanical catheter-directed thrombolysis (PCDT) to anticoagulation alone in the ATTRACT trial. Despite notable improvements in leg pain, calf circumference, and quality of life with PCDT, the primary outcome of post-thrombotic syndrome (PTS) development remains statistically indifferent between groups, prompting a reevaluation of PCDT’s overall efficacy in iliofemoral DVT.

Zooming into the femoral-popliteal subset, Kearon C et al. [31] reveal PCDT’s limited benefits in preventing PTS and its associated elevated risk of bleeding. This prompts a reconsideration of PCDT as the primary treatment for femoral-popliteal DVT.

Liu G et al. [32] introduce an alternative treatment approach with the AngioJet system, emphasizing early intervention in managing lower extremity DVT. However, their acknowledgment of study limitations underscores the necessity for further research to refine this strategy.

Weinberg I et al. [33] in a large randomized study, comparing anticoagulation alone to anticoagulation combined with percutaneous catheter-directed thrombolysis (PCDT) for acute proximal DVT, reported that PCDT is associated with lower thrombus burden at both 1 and 12 months. A thrombus-free common femoral vein (CFV) at 1 month correlates with improved clinical outcomes, while successful restoration of CFV compressibility is linked to reduced post-thrombotic syndrome (PTS) and better quality of life (QOL). However, PCDT does not significantly reduce venous valvular reflux, which appears to contribute to the progression of moderate-to-severe PTS. The study emphasizes the relationship between thrombus burden, valvular reflux, and PTS, suggesting the need for further exploration of the “open vein hypothesis” and potential alternative mechanisms influencing PTS pathophysiology.

In the comparative analysis made by Vedantham et al. [34] of AngioJet PCDT for acute proximal DVT underscores short-term benefits but emphasizes the evolving nature of DVT management, calling for continuous scrutiny and refinement.

Thukral S et al. critical analysis within the ATTRACT trial questions PCDT’s role in femoral-popliteal DVT, highlighting a lack of substantial benefits and an increased bleeding risk.

In synthesis, these studies collectively urge a departure from one-size-fits-all approaches in DVT management. The discursive exploration prompts a reflective consideration of each intervention’s nuanced impact, emphasizing the evolving landscape of DVT treatment and the imperative for individualized patient-centered care.

An exemplar study conducted by Cakir et al. [20] on percutaneous aspiration thrombectomy (PAT) revealed a substantial increase in patency rates compared to standard anticoagulant therapy. Specifically, the interventional group exhibited significantly higher patency rates at 1, 3, and 12 months, underlining the immediate impact of PAT on vascular patency.

Kasirajan et al. [21]observational study reported a 100% technical success rate using the AngioJet device, showcasing varying degrees of thrombus removal. Notably, > 90% removal was achieved in 24% of patients, 50%−90% removal in 35%, and < 50% removal in 41%, emphasizing the device’s effectiveness across a spectrum of thrombus burdens.

Loffroy et al. [22] exploration of PMT using the Aspirex®S device demonstrated a 100% technical success rate, with a primary patency rate of 90% and a secondary patency rate of 86.7% at a mean follow-up of 22.3 months. This study not only highlights the technical success of Aspirex®S but also underscores its immediate clinical success, with 90% of patients experiencing rapid recovery and discharge within 2 days after the procedure.

The ClotTriever System, as investigated by Benarroch-Gampel et al. [23] in a retrospective study involving 12 patients, showcased a 100% successful clot evacuation in a single session without the need for repeat interventions. The efficiency of the ClotTriever System is further accentuated by the short average length of hospital stay (2 days), indicative of its potential for swift patient recovery.

Wang W. et al. [28] comprehensive meta-analysis covering 35 articles and 1,323 patients spanning from 2001 to 2017 provides a holistic view of the efficacy of PMT in treating lower extremity DVT. The analysis indicates high lysis rates, low thrombosis recurrence rates, and rare severe perioperative complications associated with PMT, whether performed alone or in combination with catheter-directed thrombolysis (CDT).

Hu G et al. [29] investigation into percutaneous endovenous intervention (PEVI) compared to anticoagulation in acute LE-DVT demonstrated quantifiable reductions in post-thrombotic syndrome (PTS) and pulmonary embolism (PE) risks, increased venous patency, and a negligible increase in bleeding risk with PEVI. These numerical findings provide concrete evidence supporting the effectiveness and safety of PEVI relative to traditional anticoagulation therapy.

In summary, the incorporation of specific numerical results enriches the discourse on the efficacy and safety of mechanical thrombectomy devices in managing acute DVT. These results not only underscore the immediate and long-term benefits but also highlight the safety profiles associated with different mechanical thrombectomy approaches, offering valuable insights for healthcare professionals and researchers engaged in DVT treatment.

3. Venous stenting (Table 3).

**Table 3:** Summary of Venous stenting studies

Study	Population/Patients	Intervention	Main Outcome	Key Findings
Srinivas et al. [23]	30 consecutive patients with acute proximal lower limb DVT	Catheter-directed thrombolysis (CDT) followed by venous stenting	Immediate clinical improvement observed in all patients following venous stenting. High late patency rate observed at 12-month follow-up	Effective management of acute proximal lower limb DVT demonstrated with high late patency rate at 12-month follow-up despite complications like pulmonary embolism and stent thrombosis
AbuRahma et al. [28]	Patients with iliofemoral venous thrombosis	Conventional therapy (heparin and warfarin) vs. multimodality treatment	Significantly higher primary iliofemoral venous patency rates at 1, 3, and 5 years in the lysis/stenting group compared to conventional therapy group	Multimodality treatment (lysis/stenting) showed superior venous patency and symptom resolution rates at 30 days and 1, 3, and 5 years compared to conventional therapy
Husmann et al. [29]	Patients with acute DVT due to May-Thurner-Syndrome	Stent placement after loco-regional thrombolysis and iliac thrombectomy	Primary patency rate for venous iliac stents at 6 months follow-up was 82%. Combined surgical thrombectomy and stenting found to be safe and effective	Combined surgical thrombectomy and stenting showed technical success with complete vein patency and normal valve function. Primary patency rate for venous iliac stents at 6 months follow-up was 82%
Xue et al [35]	Patients with iliac vein compression syndrome and acute DVT	Catheter-directed thrombolysis (CDT) and stenting	5-year primary patency rates of 85.2% observed with significant improvements in pressure gradient across iliac vein stenosis	CDT for IVCS with acute DVT achieved good patency and vein function in long term
Jiang K et al [36]	74 patients with acute iliofemoral DVT	CDT plus stent placement vs. CDT alone	Superior 1-year patency rates with stent placement compared to CDT alone. Reduced venous clinical severity and improved venous Quality of Life (QoL) observed in stented patients	Stent placement post-CDT resulted in better 1-year patency rates, reduced venous clinical severity, and improved QoL compared to CDT alone
Razavi Mk et al [37]	Patients receiving stents after endovascular thrombus removal	Stent placement	Technical success reported in 94% of patients undergoing stent placement. 1-year primary patency rate after stent placement was 87%	Stent placement post-endovascular thrombus removal showed high technical success and favorable 1-year primary patency rate
Mewissen et al [38]	Stented vs. non-stented patients	Stent placement	Better 1-year patency rate observed in stented patients compared to non-stented patients	Stented patients exhibited better 1-year patency rates compared to non-stented patients, supporting the efficacy of stent placement
Thomas M. et al [39]	61 patients with acute DVT and IVCS	CDT and stent placement	Significant reductions in thigh and calf circumferences observed post-treatment. Good stent patency at 5-year follow-up	CDT and stenting led to reductions in limb circumferences and good stent patency at 5-year follow-up, indicating effectiveness in managing IVCS with acute DVT
Lilian M et al [40]	Patients undergoing iliofemoral vein stenting	Intravascular ultrasound (IVUS) examination before stent deployment	Lower 30-day stent failure requiring reintervention in IVUS + venography group. Higher 2-year primary patency rates in IVUS + venography group	IVUS utilization before stent deployment led to lower stent failure rates and higher primary patency rates, highlighting its importance in enhancing outcomes in iliofemoral vein stenting

Venous stenting serves as an additional therapy for patients experiencing acute iliofemoral deep vein thrombosis (DVT) if residual venous obstruction (RVO) persists after thrombolysis and balloon angioplasty, aiming to restore vein patency and mitigate post-thrombotic syndrome (PTS) risks. Evidence suggests a higher incidence of PTS and venous thromboembolism (VTE) recurrence when balloon angioplasty is solely employed in patients with RVO [41]. To ascertain the lesion’s nature, a combination of computed tomography or MR venogram and intravascular ultrasound (IVUS) is recommended before initiating treatment [42].

Severe PTS often results from chronic outflow obstruction, primarily involving the iliac vein, given its limited collateralization. Research indicates that when patients exhibit severe symptoms, venous stenting is warranted if the obstruction exceeds 50%, superficial collaterals develop and there is concurrent reflux in the deep and/or superficial veins. Femoropopliteal DVTs are typically managed through anticoagulation therapy alone [43].

Despite the widespread use of catheter-directed thrombolysis (CDT), there remains a dearth of compelling data, particularly concerning medium- to long-term outcomes, supporting the adjunctive use of venous stents. Chronic venous obstruction can stem from postthrombotic or nonthrombotic causes, resulting from various intrinsic, mural, and extrinsic pathologies. External compression may arise from adjacent tissues or localized compression by a pulsatile artery, exemplified by May-Thurner configurations, where the left common iliac vein is compressed by the right common iliac artery. Chronically occluded veins, often comprising collagen, pose greater treatment challenges. The outcomes are contingent upon whether the lesion is postthrombotic or nonthrombotic iliac vein lesion (NIVL), underscoring the importance of meticulously timing stenting and selecting appropriate stent designs. However, the evidence supporting intervention in NIVL cases is less conclusive, necessitating caution until more compelling data emerge [44].

Studies provide insights into the efficacy of endovascular interventions. Srinivas et al. [45] showcase promising outcomes of catheter-directed thrombolysis (CDT) and venous stenting, with immediate clinical improvement, yet complications warrant caution. AbuRahma et al. [46] emphasize the benefits of multimodality treatment involving lysis and stenting over conventional therapy, supported by Kaplan–Meier analysis. Husmann et al. [47] present a comprehensive solution for May-Thurner-Syndrome, and Xue et al. [35] highlight CDT and stenting success in iliofemoral DVT. Jiang K et al. [36] trial supports additional benefits of stent placement post-CDT, and Razavi M et al. [37] subanalysis suggests favorable long-term results. Mewissen et al. [38] registry emphasizes better 1-year patency with stent placement.

The practice of using stenting for both thrombotic and nonthrombotic deep venous pathology has become common to enhance wound healing and quality of life. Intravascular ultrasound (IVUS) examination has gained popularity for visualizing and sizing iliac vein stenoses before and after stent placement. A significant shift towards IVUS examination occurred after the Venogram vs IVUS for Diagnosing Iliac vein Obstruction (VIDIO) trial highlighted its superiority in detecting venous stenoses > 50% compared to multiplanar venography.

This study by Tran, L. M. et al. [40] aimed to evaluate the impact of adjunctive IVUS use during iliofemoral vein stenting on patency and outcomes. Data from the University of Pittsburgh Medical Center were retrospectively reviewed from January 2014 to December 2020. Patients were divided into two groups based on whether IVUS examination was used before stent deployment along with venography, compared to venography alone. Patient characteristics, procedural details, and outcomes were analyzed. The results showed that 30-day stent failure requiring reintervention was significantly lower in the IVUS + venography group compared to venography alone (10.6% vs 1.5%). Two-year primary patency rates were also significantly higher in the IVUS + venography group (90.3% vs 78.7%). IVUS utilization was found to independently protect against stent reintervention up to 2 years. Subgroup analysis revealed differences in stent characteristics based on the underlying venous disease. IVUS examination was associated with increased total stent length and stent extension below the inguinal ligament in acute DVT cases, while it was associated with larger stent diameter in NIVLs.

In conclusion, venous stenting plays a crucial role in the management of acute DVT, especially in cases of RVO after thrombolysis and balloon angioplasty. The combination of computed tomography or MR venogram with intravascular ultrasound (IVUS) aids in determining the nature of the lesion before initiating treatment, ensuring appropriate intervention. Moreover, stringent selection criteria govern the utilization of thrombolysis and venous stenting, emphasizing the importance of considering various factors such as bleeding risk, DVT anatomy, and severity of symptoms. While studies demonstrate promising outcomes of endovascular interventions, further research is warranted to establish the long-term efficacy and safety of venous stenting, particularly in cases of NIVLs. The integration of IVUS examination into clinical practice has shown significant benefits, leading to improved short-term and long-term outcomes, reduced stent failure rates, and enhanced primary patency rates, ultimately contributing to better patient care and management of venous pathology.

## Discussion

Endovascular treatment of deep vein thrombosis (DVT) has become an integral part of managing this condition, particularly in cases where anticoagulation alone may not suffice or when there is a need to alleviate symptoms promptly. One crucial aspect in the management of DVT is the timing within which to proceed with endovascular intervention. Early intervention in DVT can lead to better outcomes, including reduced risk of PTS and improve venous patency (Fig. 2).

Several studies have investigated the optimal timing for endovascular treatment of DVT, with varying conclusions based on patient characteristics, severity of thrombosis, and available resources. One notable study by Vedantham et al. [6] analyzed data from a large cohort of patients undergoing endovascular therapy for acute DVT, reporting that the average time from symptom onset to endovascular treatment ranged from 7 to 14 days, depending on the severity of symptoms and the presence of associated complications such as pulmonary embolism or limb ischemia.

However, interpreting these findings requires consideration of several factors. Firstly, the definition of “optimal timing” may vary among centers, with some focusing on symptom resolution, while others prioritize prevention of long-term complications such as post-thrombotic syndrome. Additionally, challenges in accurately determining the onset of symptoms, particularly in cases of chronic or subacute DVT, can impact the perceived urgency of intervention. Furthermore, logistical constraints within healthcare systems, such as availability of specialized interventional facilities and clinician expertise, may influence the practical feasibility of timely endovascular treatment.

The optimal timing for endovascular treatment of DVT remains a topic of debate and ongoing research within the medical community. While studies such as that by Vedantham et al. provide valuable insights into average timeframes, clinicians must individualize treatment decisions based on patient-specific factors and clinical presentation. Factors such as the presence of complications, risk of thrombus progression, and patient preferences should all be considered in determining the urgency of intervention.

The latest recommendations from the American College of Chest Physicians, the National Institute for Health and Care Excellence, and the European Society of Cardiology do not provide guidance on the utilization of intravenous stenting following catheter-directed thrombolysis (CDT) or pharmacomechanical thrombectomy for acute deep venous lesions of the lower limb, likely due to inadequate rationale. Most existing studies are retrospective, involve cohort series, or are smaller trials with varying study methodologies.

It has been evaluated in a systematic review [48] the use of venous stenting after early thrombus removal in acute iliofemoral deep vein thrombosis (DVT). It points out the limited evidence supporting its efficacy due to methodological weaknesses in existing studies, such as small sample sizes and lack of randomized controlled trials (RCTs). While some evidence suggests potential benefits in terms of maintaining venous patency and reducing post-thrombotic symptoms (PTS), uncertainties remain regarding optimal patient selection criteria and antithrombotic regimens post-stenting. The discussion underscores the need for further well-designed research to address these gaps and guide clinical decision-making effectively.

According to a systematic review by Wen-da et al. [43], which evaluated the management strategies for post-thrombotic iliac vein obstruction, venous stenting emerges as a viable treatment option in cases where the obstruction exceeds 50%. This finding underscores the importance of identifying the severity of obstruction through diagnostic imaging modalities such as venography or duplex ultrasound. Moreover, the presence of superficial collaterals further supports the indication for venous stenting. These collaterals indicate venous insufficiency and suggest a compromised venous drainage system.

Furthermore, the review suggests that the presence of reflux in both deep and/or superficial veins serves as an additional indication for venous stenting. Reflux not only exacerbates venous hypertension but also contributes to the progression of PTS symptoms. Therefore, addressing reflux through venous stenting can alleviate symptoms and potentially halt disease progression.

Conversely, femoropopliteal DVTs, which primarily involve the lower extremities, are best managed with anticoagulation therapy alone. This approach is supported by the natural history of femoropopliteal DVTs, which tend to resolve spontaneously with anticoagulation therapy, thereby minimizing the risk of recurrence and long-term complications.

Looking at future directions this study has highlighted three hot topics the use of mechanical thrombectomy devices which, with or without medical therapy, can remove even chronic thrombi, the emerging role of IVUS and the availability of new stents with adequate profiles.

Among mechanical thrombectomy devices the ClotTriever System is a mechanical thrombectomy device designed to remove large venous thrombi while minimizing trauma to the vessel wall. Abramowitz et al. [49] conducted a retrospective analysis of data from the ClotTriever Outcomes Registry, focusing on the outcomes of patients with acute and chronic lower extremity DVT, reporting rapid symptom improvement and resolution of thrombus burden. The study highlighted the feasibility and efficacy of ClotTriever treatment in patients with chronic DVT. Chronic DVT poses unique challenges due to the presence of organized thrombi and venous stenosis or occlusion. Despite these complexities, ClotTriever treatment was associated with successful thrombus removal and improvement in venous patency in a substantial proportion of chronic DVT cases.

Vedantham et al. [6] review underscores the importance of precise imaging modalities in the assessment of acute iliofemoral DVT. IVUS stands out as a valuable adjunctive tool due to its ability to provide detailed intravascular images, allowing for comprehensive evaluation of thrombus burden, composition, and vessel architecture. Unlike traditional imaging techniques such as duplex ultrasound or venography, IVUS offers direct visualization of the thrombus within the deep venous system, enabling clinicians to better characterize the extent and severity of the thrombotic occlusion.

Moreover, IVUS facilitates real-time assessment during endovascular interventions, guiding therapeutic decision-making and optimizing procedural outcomes. By accurately delineating the morphology of the thrombus, IVUS assists in determining the feasibility of various treatment approaches, including pharmacomechanical thrombolysis, catheter-directed thrombolysis, mechanical thrombectomy and venous stenting. This personalized approach to DVT management, informed by IVUS findings, enhances the efficacy of endovascular interventions while minimizing procedural risks.

Murphy E. [50] delves into the significance of four pivotal Investigational Device Exemption (IDE) trials—VIRTUS, VERNACULAR, VIVO, and ABRE—that have furnished essential insights into the safety, efficacy, and enduring consequences of iliofemoral venous stenting for obstructive disease. These trials, encompassing more than 800 patients collectively, have showcased the viability and safety of venous stenting, with minimal adverse incidents and promising patency rates observed during the 12-month follow-up period.

While these trials share similarities in their design, including enrollment criteria and patient categorization, disparities emerge in endpoint definitions and evaluation methods, particularly concerning primary patency. Differences in imaging techniques, patency criteria, and reporting protocols across the trials impede direct comparisons and underscore the necessity for standardization in forthcoming endeavors.

Initially, Wallstents were utilized in the venous system despite lacking approval for this specific indication. However, dedicated nitinol venous stents have since become available for employment in the iliofemoral venous system. Made in particular with Elgiloy material, Wallstents have now been joined by nitinol stents, which boast laser-cut designs, resulting in stents with reduced foreshortening and enhanced landing precision. This advancement has facilitated the consistent preservation of venous confluences and optimization of inflow. Moreover, nitinol stents can accommodate longer lengths required for addressing isolated compressive lesions or extensive segmental disease commonly encountered in postthrombotic venous obstruction [49].

Table 4 shows a proposed algorithmic approach to tailor individualised approach in treating acute DVT.

**Table 4:** Algorithmic approach to tailor individualised approach in treating acute DVT

1. **Anticoagulation Only**: For low-risk patients (small, distal DVTs) or those at high risk of bleeding.
**○ Anticoagulation Agents**: Use DOACs (Direct Oral Anticoagulants) or LMWH.
**○ Duration**: Short-term (3–6 months) vs. long-term anticoagulation based on recurrence risk.
2.** Catheter-Directed Thrombolysis (CDT)**: For patients with large proximal DVTs (e.g., iliofemoral) with low bleeding risk and symptoms <14 days.
**○ Thrombolytic Therapy**: tPA or urokinase delivered directly via catheter.
**○ Benefits**: Reduce post-thrombotic syndrome (PTS), increase venous patency.
**○ Logistics**: Requires availability of interventional radiology suite and skilled personnel.
3.** Pharmacomechanical Thrombectomy (PMT)**: For patients with extensive thrombus burden or those in whom CDT alone is insufficient.
**○ Devices**: Use devices such as AngioJet, Aspirex, or ClotTriever to assist in clot removal.
**○ Combination**: Often combined with CDT for better efficacy.
**○ Considerations**: Requires availability of specialized mechanical devices and operator expertise.
4.** Venous Stenting**: Consider in cases of residual venous obstruction (RVO) after CDT or PMT, especially in iliac vein compression (May-Thurner syndrome).
**○ Indications**: Obstruction >50%, development of superficial collaterals, symptomatic relief.
**○ Procedure**: Stent deployment with intravascular ultrasound (IVUS) guidance for precision.
**○ Long-Term**: Requires follow-up for patency, PTS, and complications like stent occlusion.
**Step 5: Multidisciplinary Review**
**• Team Discussion**: Involve vascular surgeons, interventional radiologists, and hematologists for consensus on complex cases.
**• Patient Preference**: Include patient in decision-making, considering their preferences, quality of life, and long-term prognosis.
**Step 6: Post-Procedure Follow-Up and Adjustment**
**• Early Follow-Up**: Within 1 month for imaging (venous duplex or IVUS) to confirm venous patency.
**• Long-Term Monitoring**: Regular follow-ups every 3–6 months for recurrence of symptoms, assessment for PTS, and anticoagulation management.

## Conclusion

The systematic review presented herein delves into the multifaceted landscape of endovascular interventions for acute deep vein thrombosis (DVT) of the lower limb, aiming to distill the wealth of evidence surrounding their efficacy, safety, and comparative outcomes. Through a meticulous synthesis of diverse studies encompassing catheter-directed thrombolysis (CDT), pharmacomechanical thrombectomy (PMT), mechanical thrombectomy, and venous stenting, this review illuminates the evolving landscape of DVT management and provides valuable insights for clinicians and researchers alike.

The amalgamation of evidence surrounding CDT underscores its promising role in enhancing venous patency and reducing the incidence of post-thrombotic syndrome (PTS) in patients with acute or subacute DVT. Noteworthy trials and systematic reviews highlight the favorable outcomes associated with CDT, emphasizing its potential to mitigate long-term morbidity while acknowledging the need for careful patient selection and risk assessment to balance benefits against bleeding risks.

In exploring pharmacomechanical thrombectomy (PMT) and mechanical thrombectomy devices, this review unveils a nuanced perspective on their efficacy and safety profiles. Studies investigating diverse mechanical thrombectomy approaches shed light on their immediate and long-term benefits, while underscoring the importance of individualized patient-centered care and continued refinement of treatment strategies.

Venous stenting emerges as a crucial adjunctive therapy in the management of acute iliofemoral DVT, particularly in cases of residual venous obstruction (RVO) post-thrombolysis and balloon angioplasty. However, the evidence surrounding its efficacy remains nuanced, necessitating further research to establish optimal patient selection criteria and long-term outcomes.

Timing and selection of endovascular interventions for DVT remain a crucial issue in the management of these patients and need to be individualized and multidisciplinary approached. While existing recommendations provide valuable guidance, the review underscores the need for further well-designed research to address existing gaps and refine treatment paradigms effectively.

In conclusion, this systematic review offers a comprehensive synthesis of the current evidence and the future prospective on endovascular interventions for acute lower limb DVT, providing clinicians and researchers with valuable insights to inform evidence-based decision-making and shape future research directions in this dynamic field.

## References

[1] Raskob GE, et al. Thrombosis: a major contributor to global disease burden. Arterioscler Thromb Vasc Biol. 2014;34(11):2363–71. 25304324 10.1161/ATVBAHA.114.304488

[2] Kim KA, Choi SY, Kim R. Endovascular treatment for lower extremity deep vein thrombosis: an overview. Korean J Radiol. 2021;22(6):931–43. 33660456 10.3348/kjr.2020.0675 PMC8154777

[3] Kearon C, et al. Antithrombotic therapy for VTE disease: CHEST guideline and expert panel report. Chest. 2016;149(2):315–52. 26867832 10.1016/j.chest.2015.11.026

[4] Di Nisio M, van Es N, Büller HR. Deep vein thrombosis and pulmonary embolism. Lancet. 2016;388(10063):3060–73. 27375038 10.1016/S0140-6736(16)30514-1

[5] Kakkos SK, et al. Editor’s Choice - European Society for Vascular Surgery (ESVS) 2021 clinical practice guidelines on the management of venous thrombosis. Eur J Vasc Endovasc Surg. 2021;61(1):9–82. 33334670 10.1016/j.ejvs.2020.09.023

[6] Vedantham S, et al. Society of interventional radiology position statement on the endovascular management of acute iliofemoral deep vein thrombosis. J Vasc Interv Radiol. 2023;34(2):284–299.e7. 36375763 10.1016/j.jvir.2022.10.038

[7] Vedantham S, et al. Pharmacomechanical catheter-directed thrombolysis for deep-vein thrombosis. N Engl J Med. 2017;377(23):2240–52. 29211671 10.1056/NEJMoa1615066 PMC5763501

[8] Baekgaard N, et al. Long-term results using catheter-directed thrombolysis in 103 lower limbs with acute iliofemoral venous thrombosis. Eur J Vasc Endovasc Surg. 2010;39(1):112–7. 19879780 10.1016/j.ejvs.2009.09.015

[9] Enden T, et al. Long-term outcome after additional catheter-directed thrombolysis versus standard treatment for acute iliofemoral deep vein thrombosis (the CaVenT study): a randomised controlled trial. Lancet. 2012;379(9810):31–8. 22172244 10.1016/S0140-6736(11)61753-4

[10] Page MJ, et al. The PRISMA 2020 statement: an updated guideline for reporting systematic reviews. Syst Rev. 2021;10(1):89. 33781348 10.1186/s13643-021-01626-4 PMC8008539

[11] Ortel TL, et al. American Society of Hematology 2020 guidelines for management of venous thromboembolism: treatment of deep vein thrombosis and pulmonary embolism. Blood Adv. 2020;4(19):4693–738. 33007077 10.1182/bloodadvances.2020001830 PMC7556153

[12] Notten P, et al. CAVA (Ultrasound-accelerated catheter-directed thrombolysis on preventing post-thrombotic syndrome) trial: long-term follow-up results. J Am Heart Assoc. 2021;10(11):e018973. 34032127 10.1161/JAHA.120.018973 PMC8483549

[13] Zhu C, et al. Comparison of clear effect and the complications, and short and mid-term effects between ultrasound-guided and non-guided catheter-directed thrombolysis in the treatment of deep venous thrombosis of lower extremity. Vascular. 2019;27(3):277–83. 30458684 10.1177/1708538118814609

[14] Engelberger RP, et al. Ultrasound-assisted versus conventional catheter-directed thrombolysis for acute iliofemoral deep vein thrombosis: 1-year follow-up data of a randomized-controlled trial. J Thromb Haemost. 2017;15(7):1351–60. 28440041 10.1111/jth.13709

[15] Zhang X, et al. A prospective randomized trial of catheter-directed thrombolysis with additional balloon dilatation for iliofemoral deep venous thrombosis: a single-center experience. Cardiovasc Intervent Radiol. 2014;37(4):958–68. 24091759 10.1007/s00270-013-0747-3

[16] Wang L, et al. Safety of catheter-directed thrombolysis for the treatment of acute lower extremity deep vein thrombosis: A systematic review and meta-analysis. Medicine (Baltimore). 2017;96(35): e7922. 28858115 10.1097/MD.0000000000007922 PMC5585509

[17] Javed A, et al. Meta-analysis of lytic catheter-based intervention for acute proximal deep vein thrombosis in the reduction of post-thrombotic syndrome. J Vasc Surg Venous Lymphat Disord. 2023;11(4):866–875.e1.37030447 10.1016/j.jvsv.2023.03.017

[18] Lu Y, et al. Catheter-directed thrombolysis versus standard anticoagulation for acute lower extremity deep vein thrombosis: a meta-analysis of clinical trials. Clin Appl Thromb Hemost. 2018;24(7):1134–43. 29132220 10.1177/1076029617739703 PMC6714738

[19] Choi YJ, et al. Comparison of treatment result between anticoagulation alone and catheter-directed thrombolysis plus anticoagulation in acute lower extremity deep vein thrombosis. Vasc Specialist Int. 2019;35(1):28–33. 30993105 10.5758/vsi.2019.35.1.28 PMC6453597

[20] Cakir V, et al. Use of percutaneous aspiration thrombectomy vs. Anticoagulation therapy to treat acute iliofemoral venous thrombosis: 1-year follow-up results of a randomised, clinical trial. Cardiovasc Intervent Radiol. 2014;37(4):969–76. 24934734 10.1007/s00270-014-0925-y

[21] Kasirajan K, Gray B, Ouriel K. Percutaneous AngioJet thrombectomy in the management of extensive deep venous thrombosis. J Vasc Interv Radiol. 2001;12(2):179–85. 11265881 10.1016/s1051-0443(07)61823-5

[22] Loffroy R, et al. Single-session percutaneous mechanical thrombectomy using the aspirex. Diagnostics (Basel). 2020;10(8):544. 32751767 10.3390/diagnostics10080544 PMC7459539

[23] Benarroch-Gampel J, et al. Technical success and short-term outcomes after treatment of lower extremity deep vein thrombosis with the ClotTriever system: A preliminary experience. J Vasc Surg Venous Lymphat Disord. 2020;8(2):174–81. 31843476 10.1016/j.jvsv.2019.10.024

[24] Crowner JR, Marston W. Percutaneous thrombectomy using a novel single-session device for acute iliocaval deep vein thrombosis. J Vasc Surg Cases Innov Tech. 2019;5(3):302–4. 31334405 10.1016/j.jvscit.2019.03.010 PMC6614594

[25] Lopez R, et al. Aspiration thrombectomy for acute iliofemoral or central deep venous thrombosis. J Vasc Surg Venous Lymphat Disord. 2019;7(2):162–8. 30639411 10.1016/j.jvsv.2018.09.015

[26] Robertson B, et al. Technical success and short-term results from mechanical thrombectomy for lower extremity iliofemoral deep vein thrombosis using a computer aided mechanical aspiration thrombectomy device. J Vasc Surg Venous Lymphat Disord. 2022;10(3):594–601. 34823046 10.1016/j.jvsv.2021.11.002

[27] Li W, et al. Effectiveness and safety of catheter-directed thrombolysis in conjunction with percutaneous mechanical thrombectomy for acute iliofemoral deep vein thrombosis: a meta-analysis. J Vasc Surg Venous Lymphat Disord. 2023;11(4):843–853.e2.10.1016/j.jvsv.2023.01.01036893883

[28] Wang W, et al. Meta-analysis and systematic review of percutaneous mechanical thrombectomy for lower extremity deep vein thrombosis. J Vasc Surg Venous Lymphat Disord. 2018;6(6):788–800. 30336908 10.1016/j.jvsv.2018.08.002

[29] Hu G, Wang J. Percutaneous endovenous intervention versus anticoagulation in the treatment of lower extremity deep vein thrombosis: a systematic review and meta-analysis. Ann Transl Med. 2022;10(18):1018. 36267764 10.21037/atm-22-4334 PMC9577808

[30] Comerota AJ, et al. Endovascular thrombus removal for acute iliofemoral deep vein thrombosis. Circulation. 2019;139(9):1162–73. 30586751 10.1161/CIRCULATIONAHA.118.037425 PMC6389417

[31] Kearon C, et al. Pharmacomechanical catheter-directed thrombolysis in acute femoral-popliteal deep vein thrombosis: analysis from a stratified randomized trial. Thromb Haemost. 2019;119(4):633–44. 30699446 10.1055/s-0039-1677795

[32] Liu G, et al. Endovascular management of extensive lower extremity acute deep vein thrombosis with AngioJet rheolytic thrombectomy plus catheter-directed thrombolysis from contralateral femoral access. Phlebology. 2019;34(4):257–65. 30049252 10.1177/0268355518790407

[33] Weinberg I, et al. Relationships between the use of pharmacomechanical catheter-directed thrombolysis, sonographic findings, and clinical outcomes in patients with acute proximal DVT: Results from the ATTRACT Multicenter Randomized Trial. Vasc Med. 2019;24(5):442–51. 31354089 10.1177/1358863X19862043 PMC6943930

[34] Vedantham S, et al. Clinical Outcomes of a pharmacomechanical catheter-directed venous thrombolysis strategy that included rheolytic thrombectomy in a multicenter randomized trial. J Vasc Interv Radiol. 2021;32(9):1296–1309.e7. 34119655 10.1016/j.jvir.2021.06.001 PMC8818274

[35] Xue GH, et al. Catheter-directed thrombolysis and stenting in the treatment of iliac vein compression syndrome with acute iliofemoral deep vein thrombosis: outcome and follow-up. Ann Vasc Surg. 2014;28(4):957–63. 24440183 10.1016/j.avsg.2013.11.012

[36] Jiang C, et al. Midterm outcome of pharmacomechanical catheter-directed thrombolysis combined with stenting for treatment of iliac vein compression syndrome with acute iliofemoral deep venous thrombosis. J Vasc Surg Venous Lymphat Disord. 2020;8(1):24–30. 31327743 10.1016/j.jvsv.2019.03.020

[37] Razavi M, et al. The initial report on 1-year outcomes of the feasibility study of the VENITI VICI VENOUS STENT in symptomatic iliofemoral venous obstruction. J Vasc Surg Venous Lymphat Disord. 2018;6(2):192–200. 29290601 10.1016/j.jvsv.2017.10.014

[38] Mewissen MW, et al. Catheter-directed thrombolysis for lower extremity deep venous thrombosis: report of a national multicenter registry. Radiology. 1999;211(1):39–49. 10189452 10.1148/radiology.211.1.r99ap4739

[39] Medjeral-Thomas N, et al. Retrospective analysis of a novel regimen for the prevention of venous thromboembolism in nephrotic syndrome. Clin J Am Soc Nephrol. 2014;9(3):478–83. 10.2215/CJN.07190713. 24334865 10.2215/CJN.07190713 PMC3944768

[40] Lilian M, et al. Intravascular ultrasound evaluation during iliofemoral venous stenting is associated with improved midterm patency outcomes. J Vasc Surg Venous Lymphat Disord. 2022;10(6):1294–303. 10.1016/j.jvsv.2022.05. 35872140 10.1016/j.jvsv.2022.05.016

[41] Comerota AJ, et al. Postthrombotic morbidity correlates with residual thrombus following catheter-directed thrombolysis for iliofemoral deep vein thrombosis. J Vasc Surg. 2012;55(3):768–73. 22277690 10.1016/j.jvs.2011.10.032

[42] Gagne PJ, et al. Analysis of threshold stenosis by multiplanar venogram and intravascular ultrasound examination for predicting clinical improvement after iliofemoral vein stenting in the VIDIO trial. J Vasc Surg Venous Lymphat Disord. 2018;6(1):48–56.e1. 29033314 10.1016/j.jvsv.2017.07.009

[43] Wen-da W, Yu Z, Yue-Xin C. Stenting for chronic obstructive venous disease: a current comprehensive meta-analysis and systematic review. Phlebology. 2016;31(6):376–89. 26205370 10.1177/0268355515596474

[44] Breen K. Role of venous stenting for venous thromboembolism. Hematology Am Soc Hematol Educ Program. 2020;2020(1):606–11. 33275696 10.1182/hematology.2020000147 PMC7727585

[45] Srinivas BC, et al. Outcome of venous stenting following catheter directed thrombolysis for acute proximal lower limb venous thrombosis: a prospective study with venous Doppler follow-up at 1-year. Cardiovasc Interv Ther. 2015;30(4):320–6. 25708707 10.1007/s12928-015-0317-5

[46] AbuRahma AF, et al. Iliofemoral deep vein thrombosis: conventional therapy versus lysis and percutaneous transluminal angioplasty and stenting. Ann Surg. 2001;233(6):752–60. 11371733 10.1097/00000658-200106000-00004 PMC1421317

[47] Husmann MJ, et al. Stenting of common iliac vein obstructions combined with regional thrombolysis and thrombectomy in acute deep vein thrombosis. Eur J Vasc Endovasc Surg. 2007;34(1):87–91. 17324594 10.1016/j.ejvs.2007.01.006

[48] Taha MA, et al. A systematic review on the use of deep venous stenting for acute venous thrombosis of the lower limb. Phlebology. 2019;34(2):115–27. 29788818 10.1177/0268355518772760

[49] Abramowitz SD, et al. Six-month deep vein thrombosis outcomes by chronicity: analysis of the real-world clottriever outcomes registry. J Vasc Interv Radiol. 2023;34(5):879–887.e4. 37105663 10.1016/j.jvir.2022.12.480

[50] Murphy EH. Venous stenting: a behind-the-scenes look at the trial data. In Endovascular today; 2022. p. 6.

